# Development and Validation of a Framework for Smart Wireless Strain and Acceleration Sensing

**DOI:** 10.3390/s22051998

**Published:** 2022-03-03

**Authors:** Omobolaji Lawal, Amirali Najafi, Tu Hoang, Shaik Althaf V. Shajihan, Kirill Mechitov, Billie F. Spencer

**Affiliations:** Department of Civil and Environmental Engineering, University of Illinois, 205 N. Matthews Ave, Urbana, IL 61801, USA; oglawal2@illinois.edu (O.L.); anajafi2@illinois.edu (A.N.); tuhoang2@illinois.edu (T.H.); sav4@illinois.edu (S.A.V.S.); mechitov@illinois.edu (K.M.)

**Keywords:** strain sensor, structural health monitoring, multimetric sensing, framework, wireless smart sensor

## Abstract

Civil infrastructure worldwide is subject to factors such as aging and deterioration. Structural health monitoring (SHM) can be used to assess the impact of these processes on structural performance. SHM demands have evolved from routine monitoring to real-time and autonomous assessment. One of the frontiers in achieving effective SHM systems has been the use of wireless smart sensors (WSSs), which are attractive compared to wired sensors, due to their flexibility of use, lower costs, and ease of long-term deployment. Most WSSs use accelerometers to collect global dynamic vibration data. However, obtaining local behaviors in a structure using measurands such as strain may also be desirable. While wireless strain sensors have previously been developed by some researchers, there is still a need for a high sensitivity wireless strain sensor that fully meets the general demands for monitoring large-scale civil infrastructure. In this paper, a framework for synchronized wireless high-fidelity acceleration and strain sensing, which is commonly termed multimetric sensing in the literature, is proposed. The framework is implemented on the Xnode, a next-generation wireless smart sensor platform, and integrates with the strain sensor for strain acquisition. An application of the multimetric sensing framework is illustrated for total displacement estimation. Finally, the potential of the proposed framework integrated with vision-based measurement systems for multi-point displacement estimation with camera-motion compensation is demonstrated. The proposed approach is verified experimentally, showing the potential of the developed framework for various SHM applications.

## 1. Introduction

Civil infrastructure plays a vital role in societal activities and economic development. Indeed, many developed nations worldwide have invested heavily in such infrastructure. For instance, the United States recently introduced a new USD 1 trillion infrastructure bill to construct and maintain existing civil infrastructure. However, such infrastructure is subject to factors such as aging and deterioration. Therefore, proper maintenance is critical to ensuring safe and reliable operation; lack of adequate maintenance can lead to structural degradation that may result in failure and collapse. Examples of poorly maintained civil infrastructure leading to catastrophic failure resulting in loss of lives and assets can be found all over the world, including the collapses of the FIU pedestrian bridge [1], the surfside condo in Miami [2], the Suzhou hotel in China [3], and an apartment building in Cairo [4], which resulted in 6, 98, 17, and 25 deaths, respectively. These events illustrate the need to properly maintain and monitor civil infrastructure.

Visual inspection is the primary approach for assessing a structure’s condition and ensuring proper maintenance [5]. However, this approach has many challenges, including human error and the inability to offer continuous condition assessment. To overcome these challenges, researchers have proposed the use of structural health monitoring (SHM) to achieve early detection of damage in structures, to enable on-demand maintenance decisions, and, ultimately, to prevent catastrophic collapse [6]. Conventional structural health monitoring systems use wired sensors that have been widely adopted in practice [7,8,9]. However, this wired SHM systems can be labor intensive and costly.

Recent advances in SHM using wireless smart sensor (WSS) technologies offer attractive alternatives to wired sensors due to their relative ease of installation and lower maintenance costs [10]. This low cost also allows dense arrays of sensors to be deployed. Results using WSSs have been promising, as they provide rich information using their on-board processing and wireless communication capabilities. WSSs eliminate the need for cables through their wireless connection to a central unit, thereby allowing for fast deployment and flexibility of use [11,12,13,14]. With these technologies, the state of a structure can be assessed more autonomously, efficiently, and on-demand.

Most of the available WSSs only make use of accelerometers to measure structural vibrations, primarily because a reference is not required, resulting in ease of installation. The modal characteristics can be extracted from measured acceleration data that can inform the global condition of a structure. For example, Lynch et al. [15] developed a WSN making use of MEMS accelerometers for monitoring structures. Kim et al. [16] designed an accelerometer sensor board for the MicaZ platform deployed on the Golden Gate Bridge. Jo et al. [17] developed a high sensitivity accelerometer board to measure low level ambient vibration using the commercial Imote2 platform. Bedon et al. [18] proposed and validated the use of a MEMS accelerometer sensor prototype for SHM tasks. Zhu et al. [19] developed a high-resolution accelerometer for the Xnode platform. The Xnode is a next-generation WSS platform developed by the Smart Structures Technology Laboratory (SSTL) at the University of Illinois at Urbana-Champaign. Fu et al. [20] validated the hardware and software design of the Xnode platform through full-scale field testing on a suspension bridge. The testing demonstrated that the Xnode is robust, capable of high-fidelity measurements, and efficient for long-term monitoring. Additionally, V. Shajihan et al. [21] integrated an extremely high sensitivity WSS-accelerometer into the Xnode platform. The Xnode software enables synchronized data acquisition using multiple sensor nodes with microsecond accuracy. However, even such high-sensitivity accelerometers are unable capture the pseudo-static response of a structure.

While acceleration is viewed as a global measurand, strain, on the other hand, can accurately measure local behaviors, and is also excellent for measuring low frequency motion. Some researchers have previously developed WSSs for measuring strain. For example, Nagayama et al. [22] developed a strain sensor board for the Berkeley mote WSS for civil engineering applications. Choi et al. [23] developed a prototype strain sensor board for the Mica2 mote. Whelan et al. [24] designed a signal conditional board for the Tmote Sky WSN platform. Jo et al. [25] designed a strain sensor board for the Imote2 platform to measure low-level ambient strains. Liu et al. [26] developed a multichannel strain gage signal conditioner for measuring dynamic strain using the IRIS mote platform. However, the resolution and dynamic range for these boards are limited. Won et al. [27] recently developed a multimetric sensor board for strain and acceleration using the Xnode platform. While the board allows for high fidelity multimetric sensing, general application is limited due to the strain and acceleration sensors being placed on a single board, which may result in power consumption issues. Note that strain gage signal conditioner integrated circuits (ICs) are also commercially available. However, taking the PGA 900 IC as an example, while it possesses the basic features described herein, it also has an in-built microcontroller unit, thus has a higher cost and power consumption. Despite these developments, there is still a need for a high sensitivity wireless strain sensor that fully meets the demands for monitoring large-scale civil infrastructure.

In this paper, a framework for multimetric sensing of strain and acceleration is developed using the Xnode wireless sensing platform. The main contributions of this work are: (i) development of strain acquisition on a wireless sensor platform to integrate with acceleration measurements; (ii) multimetric displacement estimation and its use for camera motion compensation without needing a stationary reference using the integrate wireless platform; and (iii) evaluation under real world practical conditions. This work builds on the existing Xnode sensing framework developed by researchers at SSTL. However, this paper differs from recent works done by the research group [28,29,30] in that a multimetric sensing framework is implemented. Additionally, a WSS is utilized for improving camera measurement results.

The rest of the article is organized as follows: First, Section 2 details the strain sensor board design. Subsequently, in Section 3, the software developed to combine the acquisition of strain with the Xnode’s accelerometer measurements is described. This combination provides the Xnode with effective high fidelity multimetric sensing capabilities. In Section 4, laboratory experiments are carried out to validate the performance of the designed strain sensor. The efficacy of this sensing framework is demonstrated in Section 5 through the displacement of a two-story structure in the laboratory. The displacements are estimated by using a Kalman filter with the multimetric data. Furthermore, in Section 6, the framework is integrated with a camera-measurement system by leveraging the displacements estimated through the multimetric approach to compensate for camera motion errors that may occur when carrying out vision-based measurements to obtain multi-point displacement estimates. Finally, Section 7 concludes the paper, and the results show the proposed framework enables high fidelity multimetric sensing of strain and acceleration.

## 2. Development of Wireless Strain Sensor Board

This section describes the key features of the hardware design for the proposed framework’s strain sensor and its interface to the Xnode. Following a brief description of the Wheatstone bridge commonly employed to sense strain, the features of the hardware design are described, leveraging the Xnode’s modular architecture and high-resolution analog to digital converter (ADC), as well as building upon the work of Jo et al. [25] for the Imote2 platform.

### 2.1. Wheatstone Bridge

**Figure 1:**
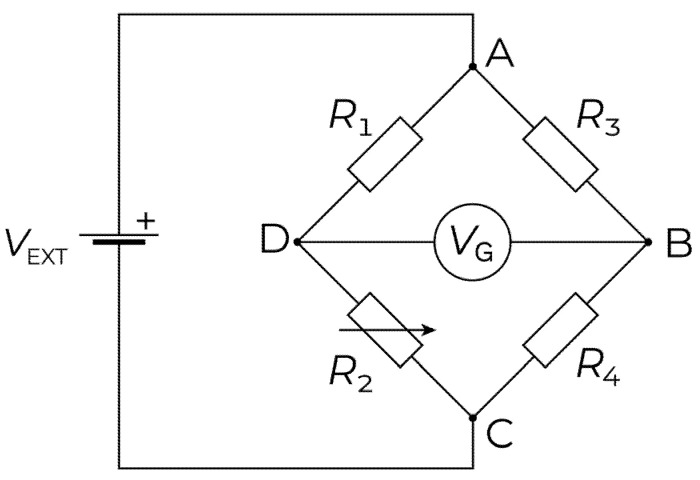
Wheatstone bridge circuit diagram.

A Wheatstone bridge is an electrical circuit used for measuring resistance [31]. The bridge consists of two arms of resistors connected in parallel with an external voltage applied across the circuit, as shown in Figure 1. The resistances, $R_{1},\;R_{3},\;R_{4}$, are electrical resistors, $R_{2}$ is the strain gage’s resistance, $V_{ext}$ is the external power supply, and $V_{G}$ is the output voltage (i.e., voltage between points B and D), respectively.

The output from the Wheatstone bridge circuit can be determined from:

(1) $$V_{G}=V_{ext}\left[\frac{R_{1}}{R_{1}+R_{2}}-\frac{R_{3}}{R_{3}+R_{4}}\right]$$

Consider a balanced circuit i.e., $R_{1}=R_{3}=R_{4}=R$; then, if $R_{2}=R$ when no load is applied (i.e., ε=0), the result will be $V_{G}=0$. If, subsequently, a change in strain occurs, the resistance in the strain gage will become $R_{2}=R+\Delta R$, and the output voltage from the Wheatstone bridge is expressed as:

(2) $$V_{G}=V_{ext}\left[\frac{-\Delta R}{4R+2\Delta R}\right]$$

The strain ε is given by:

(3) $$\varepsilon =V_{ext}\left[\frac{-4V_{G}}{GF\times V_{ext}}\right]$$

where GF=(ΔR/R/ε) is the gage factor. However, variation is always found in the resistance of the resistors in the Wheatstone bridge, as well as in the resistance $R_{2}$ of the unloaded strain gage. These variations will lead to unequal resistors in the circuit, resulting in the output voltage being:

(4) $$V_{G}=V_{ext}\left[\frac{R}{2R+\Delta R_{1}+\Delta R_{2}}-\frac{R}{2R+\Delta R_{3}+\Delta R_{4}}\right]$$

which is nonzero, even though no load is applied.

Depending on the application and the ADC type, several hundred to thousands of times amplification gain are often required [25]. If the nonzero offset due to imbalance given in Equation (4) is amplified using such high gains, the voltage can exceed the measurable range of the ADC, resulting in saturation. In such cases, the bridge must be balanced before amplification. As described in the sequel, the proposed hardware design addresses this problem by (a) precisely balancing the circuit using digital potentiometers, and (b) utilizing the high resolution 24-bit ADC of the Xnode to reduce the level of signal amplification required.

### 2.2. Precisely Balanced Wheatstone Bridge and Signal Amplification

**Figure 2:**
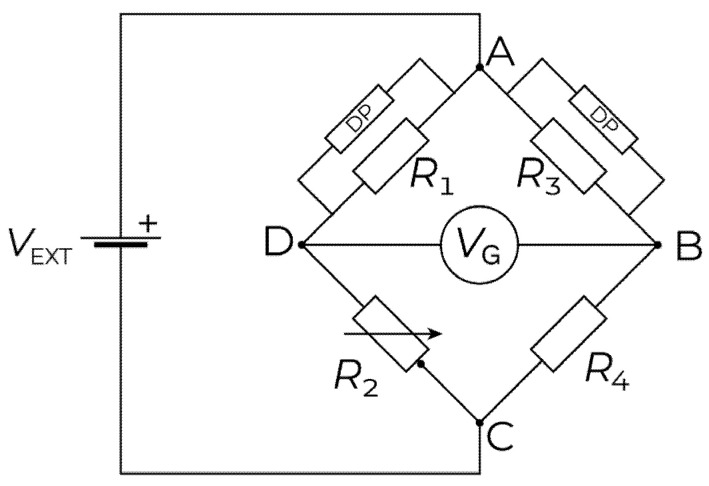
Wheatstone bridge with digital potentiometers.

To precisely balance of the Wheatstone bridge automatically prior to amplification, two digitals (DP) are added, as shown in Figure 2. The potentiometer employed is the TPL0102 from Texas Instruments, which has two channels, each with 256 levels of resistance, ranging from 0 to 100 kΩ. The DPs are used to adjust the resistance values, such that the bridge circuit is precisely balanced (i.e., for zero applied strain, the output voltage is $V_{G}=0$). The DPs resistance level which balances the bridge is stored in a non-volatile memory so that the balanced condition is preserved when power is turned off.

For this research, signal amplification is achieved using MAX4194 from Maxim Integrated, which is a variable gain precision instrumentation amplifier. The gain is given by:

(5) $$g=1+\frac{50,000}{R_{G}}$$

where g is the amplifier’s gain, and $R_{G}$ is the resistor that determines the amplifier’s gain. The constant 50,000 in Equation (5) corresponds to the value of the sum of the two resistors internally connected to the input operational amplifiers in the MAX4194 amplifier. The high resolution of the 24-bit ADC employed by the Xnode obviates the need for extremely high levels of signal amplification. Thus, in this design, two 2 kΩ and one 1 kΩ are connected in parallel using jumper switches, achieving four possible amplification gains, i.e., 26, 51, 76, and 101. In addition, the Xnode’s ADC has internal software selectable amplification gains of 1, 2, 4, 8, and 12, which, when combined, with the MAX4194 amplifier, allows for amplification of the output from the Wheatstone bridge ranging from 1 to 1212 times.

### 2.3. Shunt Calibration and Temperature Compensation

Sensor calibration is an essential step in ensuring measurement accuracy. Since site conditions differ from those in the laboratory, a calibration performed in the laboratory might not be applicable in a field setting. Therefore, the designed sensor accommodates an on-board calibration feature. The shunt calibration process involves applying a predetermined strain input to the Wheatstone bridge circuit. Shunt calibration is achieved by decreasing the arm of the bridge with the active strain gage through the parallel connection of a larger resistor with a known value. The output of the Wheatstone bridge circuit is then compared with the predetermined strain to verify the circuit accuracy and obtain the sensitivity of the circuit. The sensitivity value obtained is then used to convert measured voltage to physical strain. Herein, a 100 kΩ shunt resistor controlled by a digital switch is used. This corresponds to a predetermined input of 1668.8 με (using GF = 2.09).

Most strain gages experience thermally induced strain due to environmental temperature changes. For long-term field measurements, this can be a big concern, as the thermal effects lead to errors in the true strain measured. In this, two strain gages in the same bridge and mounted at the same location undergo similar temperature changes; hence the ratio of resistance to output voltage remains unchanged. Thus, the use of a dummy gage is a common way to overcome this problem. Therefore, the developed strain sensor provides a user-selectable configuration where resistor $R_{4}$ (Figure 2) can be replaced with a dummy gauge. The selection is controlled by an analog switch.

### 2.4. Integration with the Xnode Platform

**Figure 3:**
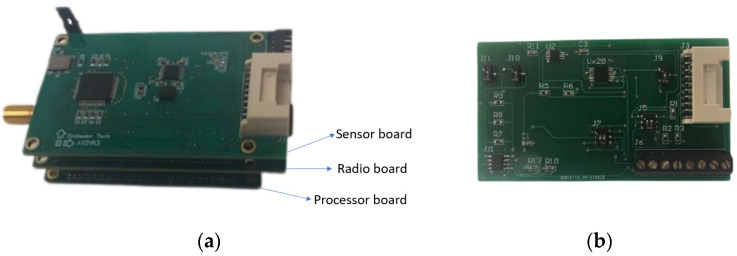
Xnode Smart Sensor. (**a**) Original Xnode platform. (**b**) Newly designed strain sensor extension.

The strain sensor board is designed to be connected to the A101-R3 sensor board on the Xnode using a 20-pin connector cable. The Xnode’s modular architecture allows for possible connections to external sensors through a 20-pin connector. The 20-pin connector provides access to five of the eight channels of the Xnode’s ADC; the remaining three channels for the ADC are allocated to for the triaxial accelerometer. [20]. A power supply, ground, GPIO, and I^2^C pins are also exposed on the 20-pin connector. From the hardware perspective, the power and ground pins on the 20-pin connector are routed to provide excitation the Wheatstone bridge for the strain sensor. Through the connection of the I^2^C pins between the strain sensor and the 20-pin connector, the balancing of the Wheatstone bridge before amplification is controlled digitally from the Xnode software. Subsequently, the output from the Wheatstone bridge is routed to a fourth channel on the Xnode’s 20-pin connector. Additionally, the GPIO pin present is used to connect the shunt resistor on the strain sensor to allow for shunt calibration. This integration allows the use of the Xnode’s 24-bit ADC, and enables synchronized multimetric sensing. The detailed overview of the Xnode is shown in Figure 3a and the designed strain sensor board is shown in Figure 3b.

## 3. Software Implementation for Multimetric Sensing Framework

This section provides an overview for the software design for the proposed framework. The Xnode makes use of an open-source real-time operating system, FreeRTOS, implementing key middleware infrastructure allowing synchronized sensing and reliable communication [20]. The software designed for strain sensing is integrated with the existing Xnode software to enable multimetric sensing.

To provide synchronized wireless data collection for SHM field applications, the Xnode software uses the application, “*RemoteSensing*” [20,21]. For strain channels, the automated bridge balancing and shunt calibration features are integrated with the *RemoteSensing* application to allow effective multimetric sensing. Before performing signal amplification, the Wheatstone bridge is balanced automatically by adjusting the wiper locations of the DPs across both arms of the bridge and comparing the output signal to a threshold value. The resistances of bridge arms are adjusted until the output signal is less than the threshold value.

**Figure 4:**
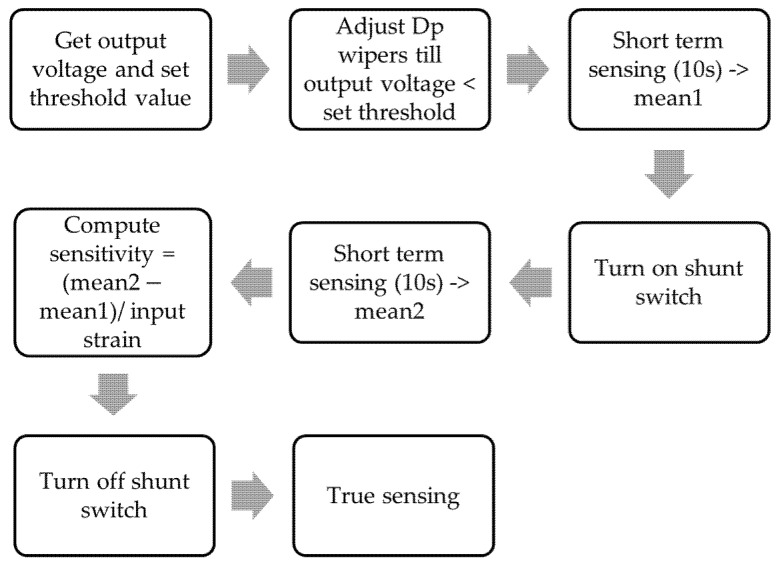
Diagram of software implemented autobalancing and shunt calibration process.

After the automated balancing is completed, shunt calibration can be carried out. The shunt resistor is connected to a digital switch, which simulates a known input strain when turned on. Subsequently, the sensitivity is obtained as the ratio between the output voltage and the predetermined strain. Finally, the shunt switch is turned off and sensing can then be carried out. The obtained sensitivity value is used to convert the measured voltage to strain. Figure 4 shows a simplified breakdown of the automated bridge balancing and shunt calibration algorithm.

**Figure 5:**
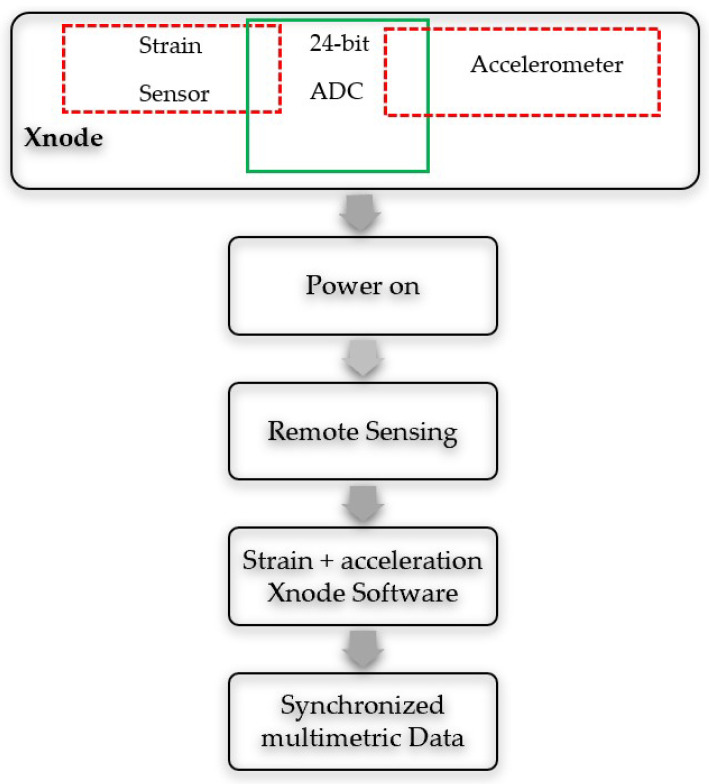
Flowchart of proposed multimetric sensing framework.

The integration of the strain sensing features onto the Xnode platform allows synchronized multimetric sensing to be developed. The proposed framework for multimetric synchronized sensing of strain and acceleration is illustrated in the flowchart shown in Figure 5.

## 4. Experimental Validation

In this section, the accuracy of the strain sensor hardware and software developed for the Xnode platform is verified through laboratory experiments for both static and dynamic strain measurements. The static strain is verified by using a cantilever beam with known loads placed at the free end. For the dynamic strain verification, the free vibration response of a two-story test structure is measured for an input impulse load.

### 4.1. Validation for Static Strain Measurements

**Figure 6:**
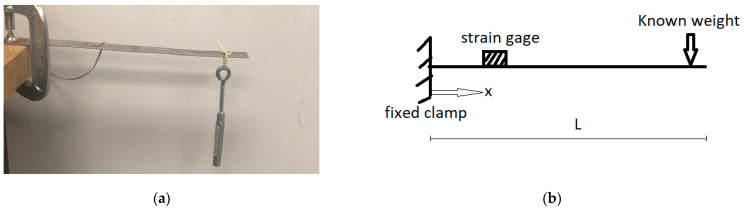
Static strain verification test set up. (**a**) Physical set up. (**b**) Schematic.

The accuracy of the strain sensor for static loading is verified by comparing measurements for a simple cantilever beam set up in the laboratory, illustrated in Figure 6a, with the theoretically obtained values. A schematic of the loading setup is shown in Figure 6b. Table 1 lists the structural properties used in the experiment.

The moment at any location in a cantilever beam subject to a point load at the free end can be theoretically estimated. Moreover, the bending stress at that location can be estimated using the flexure formula:

(6) $$\;\varepsilon =\frac{\sigma }{E}=\frac{\mathrm{My}}{\mathrm{EI}}$$

where σ, M, y, I, and E represent bending stress, bending moment, depth of neutral axis, moment of inertia, and modulus of elasticity, respectively. Note that the above expression is only valid for thin and slender beams. In general, many bridges can be well-approximated as Euler–Bernoulli beams [32]; the challenge comes in determining the exact location of the neutral axis, which will affect the results from using Equation (6). However, with appropriate analysis, the neutral axis can be determined with sufficient accuracy.

For this test, the steel beam is loaded with predetermined weights at the free end, as shown in Figure 6a. In total, three different weights were applied, and the strains were theoretically estimated to be 35.92 με, 62.85 με, and 134.7 με respectively. The corresponding measured strains to each of the applied weights were obtained as 36.51 με, 63.21 με, and 136 με respectively, using the Xnode strain sensor. Table 2 summarizes the results from the static strain validation tests. The theoretical values are seen to be in good agreement with the experimental results, thereby illustrating the developed strain sensor’s capabilities in measuring static strains.

### 4.2. Validation for Dynamic Strain Measurements

**Figure 7:**
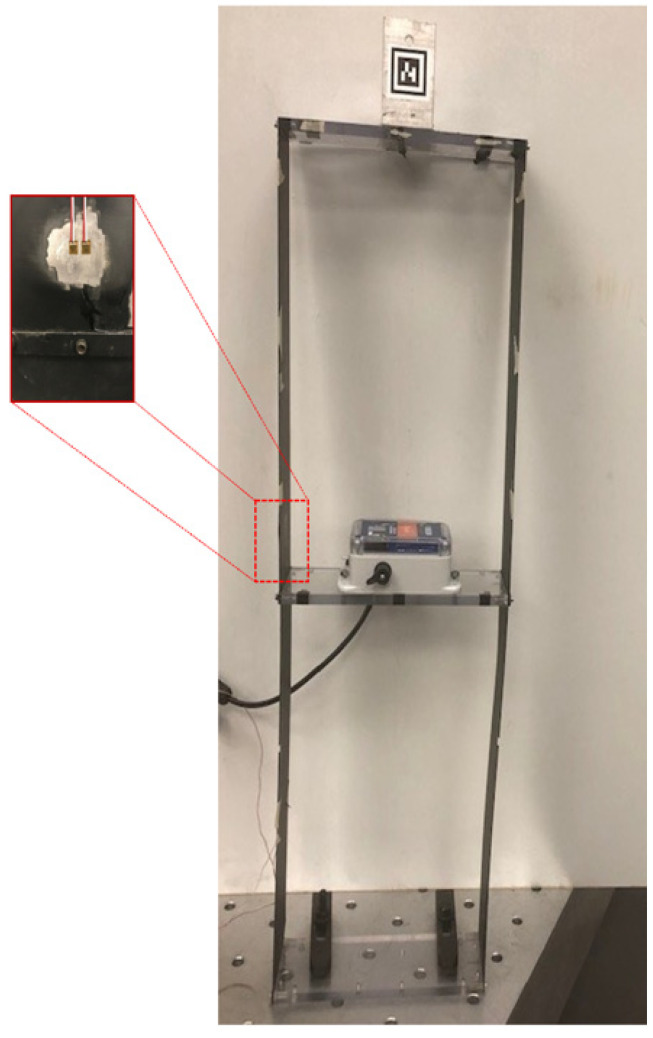
Two-story shear frame for dynamic strain verification test. The inset shows the 350 Ω employed for the study.

For verification of dynamic strain measurement, the one-bay two-story shear frame shown in Figure 7 is used. The test structure has natural frequencies at 1.7 Hz and 4.2 Hz The dynamic strain measured by the wireless Xnode strain sensor is verified using measurements from a Micro-Measurements 2100 Signal Conditioning Amplifier [33], calibrated such that 1 mV represents 1 με. A DS1054Z RIGOL digital oscilloscope was used for visualization of the acquired strain signals. For this verification, two 350 Ω foil type strain gages with gauge factors of 2.09 are used, each connected respectively to the wired and wireless strain sensing systems. A 100 Hz sampling rate is used in both the cases.

**Figure 8:**
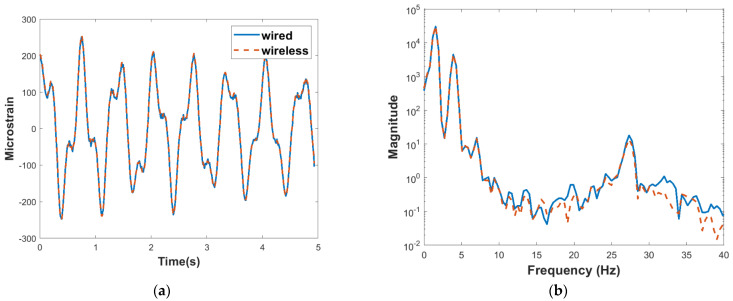
Dynamic strain verification. (**a**) Time domain comparison of strain response of wireless and wired sensing systems. (**b**) Power spectral densities of wireless and wired strain responses.

An impulse load was applied at the top story in the transverse direction of the building structure, and the free vibration response was measured. The strain response was measured for approximately 5 s, and the results obtained in the time domain are shown in Figure 8a. The frequency domain representation is shown in Figure 8b. For this comparison, cross-correlation was used to synchronize the measurements from the two sensing systems [34]. The results obtained show a good agreement between the wired and wireless systems.

With the static and dynamic performance of the strain sensor confirmed, the next sections turn to some applications. First, the strain sensor is used in multimetric displacement estimation, and then camera motion compensation using the estimated multimetric displacement is illustrated.

## 5. Application: Multimetric Displacement Estimation

This section explores the application of the designed smart strain sensor for displacement estimation, where both strain and acceleration are used as inputs. Displacement is an important quantity of interest in SHM, due to its wide use in limit design checks. Displacement sensors, such as linear variable differential transducers, are readily available commercially; however, these sensors require reference points that are often unavailable in full-scale applications. As such, measured acceleration has been commonly used as an indirect displacement estimation method because no reference is required. Although obtaining displacement from acceleration can theoretically be achieved through double integration, significant errors (e.g., drift) can arise due to the high-noise floor in accelerometers in the low frequency range [35]. Alternatively, strain is another indirect method that has also been used for displacement estimation by researchers. For example, Chang and Kim [36] proposed a method for estimating dynamic displacements from measured strain using empirical mode decomposition. Kang et al. [37] introduced an approach for estimating dynamic displacements based on the modal strain-displacement relationship. The mode shapes were obtained from the finite element model of the test structure. In general, using acceleration for displacement estimation is reliable for dynamic displacements but suffers from errors in the low frequency regions. On the other hand, strain provides accurate data at low frequencies, but are unsuitable for higher frequency measurements due to limits in resolution and high noise-to-signal ratios [38]. Therefore, using these indirect methods for displacement estimation can still be problematic.

Park et al. [39] proposed an algorithm for fusion of strain and acceleration to obtain more accurate structural displacement estimates. This algorithm was extended using synchronized strain and acceleration measurements [40]. However, the proposed method was limited to simply supported bridge structures, for which the response could be represented spatially as a sinusoid. Subsequently, Cho et al. [41,42] extended the approach for use with continuous beam-type structures, first by using repeated sinusoidal functions for the mode shapes, and then by incorporating mode shapes from finite element models to account for more general types of bridges. The underlying advantage of multimetric sensing lies in the leveraging of the accurate strain data obtained in the low-frequency region with accelerations in higher frequency regions to obtain optimal displacement estimates.

The Kalman filter has also been used to fuse data from multiple types of sensors for displacement estimation. For example, Smyth et al. [43] used the Kalman filter to combine collocated acceleration and GPS-based displacement measurements to obtain more precise dynamic displacements. Cho et al. [44] modified the Kalman filter for data fusion of strain and acceleration measurement to estimate the displacement for bridges. Kim et al. [45] used a Kalman filter that includes acceleration bias by incorporating the error due to integration in its state–space model to combine acceleration and intermittent displacement data from a laser sensor for displacement estimation. Zhu et al. [46] used a multi-rate Kalman filter approach to combine acceleration and strain data to estimate displacements in tall, slender structures. Sarwar et al. [47] applied an adaptive Kalman filter to fuse collocated acceleration and strain measurements to estimate bridge displacements. Kalman filter-based data fusion has been widely employed for displacement estimation, and will be used for the application discussed herein. It should be noted that the use of Kalman filter for the proposed approach is not computationally expensive, as only the first few modes are typically considered in civil engineering structures. Nevertheless, a wide variety of algorithms could also be considered other than the Kalman filter. For example, the recursive principal component analysis (PCA) has recently been employed for real-time structural health monitoring applications [48]. Comparisons were made with the recursive PCA in terms of computational costs. For this case, the same test structure shown in Figure 7 of the revised manuscript was used. The first-floor acceleration response is measured from an input transverse impulse at the top of the structure. The measured acceleration is then used as an input to both the Kalman filter and recursive PCA for obtaining the states. The process using the Kalman Filter was computed in 0.08 secs, while the computation was performed in 0.05 secs using the recursive PCA algorithm. The obtained states can then be used with displacement from strain to achieve towards achieving multimetric displacement estimates. For ease of evaluation, the computation was carried out on Matlab using an intel core i7-6600U CPU @ 2.6 GHz processor. The recursive PCA was faster, although the computational time for Kalman Filter is also within a comparable range.

The above shows the need for a multimetric-based displacement estimation. Additionally, there are currently no available algorithms that provide both static and dynamic displacement estimates with a single sensor. For example, Gomez, et al. [49] proposed an approach to estimate dynamic displacements from a single accelerometer sensor and showed good performance above 1 Hz; displacements below 1 Hz could not be captured. On the other hand, while Park et al. [39] has shown that low-frequency displacements can be estimated from a single strain sensor: the performance of this approach above 7.5 Hz is poor, due to high signal-to-noise in this frequency region. The adopted algorithm is described in the following subsection.

### 5.1. Kalman Filter-Based Displacement Estimation

This paper uses the Kalman filter data fusion approach described by Cho et al. [44], a summary of which is described in this section for the convenience of the reader. First, a state–space model is developed with acceleration as the input and displacement as output, i.e.:

(7) $$\left[\begin{array}{c} \dot{x}\left(t\right)\\ \ddot{x}\left(t\right) \end{array}\right]=\left[\begin{array}{cc} 0 & 1\\ 0 & 0 \end{array}\right]\left[\begin{array}{c} x\left(t\right)\\ \dot{x}\left(t\right) \end{array}\right]+\left[\begin{array}{c} 0\\ 1 \end{array}\right]a\left(t\right)+w\left(t\right)\;\dot{x}\left(t\right)=\mathrm{Ax}+Ba\left(t\right)+w\left(t\right)$$

(8) $$u\left(t\right)=\left[\begin{array}{cc} 1 & 0 \end{array}\right]\left[\begin{array}{c} x\left(t\right)\\ \dot{x}\left(t\right) \end{array}\right]+v\left(t\right)\;=\mathrm{Cx}+v{\left(t\right)}_{\;}$$

where x(t), a(t), u(t), w(t), and v(t) are the state vector, acceleration, measured displacement, the process noise, and measurement noise, respectively. Note that the above state–space representation makes use of measured signals from sensors and does not depend on structural parameters. However, the overall model can be affected by the mode shapes used, as will be shown in the next section. For a multi-degree of freedom civil engineering structure, the response of the structure is usually characterized by only the first few modes [50]. Therefore, the proposed method can be considered robust to parameter uncertainties.

Since the recorded signals are discrete in nature, Equations (7) and (8) are discretized assuming a piecewise linear input and is given by:

(9) $$x\left[n+1\right]=\left[\begin{array}{cc} 1 & dt\\ 0 & 1 \end{array}\right]x\left[n\right]+\left[\begin{array}{c} \frac{{\left(dt\right)}^{2}}{2}\\ dt \end{array}\right]a\left[n\right]+w\left[n\right]\;=Ax\left[n\right]+Ba\left[n\right]+w{\left[n\right]}_{\;}$$

(10) $$u\left[n\right]=\left[\begin{array}{cc} 1 & 0 \end{array}\right]x\left[n\right]+v\left[n\right]\;=Cx\left[n\right]+v{\left[n\right]}_{\;}$$

where n is the discrete time index, and *dt* is the sampling time.

The Kalman filter model is based on using the measured acceleration for the time update step given by:

(11) $$\hat{x}\left[n+1|n\right]=A\hat{x}\left[n|n\right]+Ba\left[n\right]$$

(12) $$P\left[n+1|n\right]=AP\left[n|n\right]A^{T}+Q$$

where $\hat{x}$ is the state estimate, and P is error covariance matrices. Here, Q and R are the noise covariance matrices given by:

(13) $$Q=q\left[\begin{array}{cc} \frac{{\left(dt\right)}^{3}}{3} & \frac{{\left(dt\right)}^{2}}{2}\\ \frac{{\left(dt\right)}^{2}}{2} & dt \end{array}\right]$$

(14) $$R=\frac{r}{dt}$$

where q and r are covariances of Gaussian random processes.

Then, the displacement $\bar{u}\left[n\right]$ derived from the measured strain is used together with the Kalman gain estimated for the measurement update:

(15) $$\hat{x}\left[n|n\right]=\hat{x}\left[n|n-1\right]+M_{x}\left[n\right]\left(\alpha \bar{u}\left[n\right]-C\hat{x}\left[n|n-1\right]\right)$$

(16) $$P\left[n|n\right]=\left(I-M_{x}\left[n\right]C\right)P\left[n|n-1\right]$$

(17) $$\hat{u}\left[n|n\right]=C\hat{x}\left[n|n\right]$$

where $M_{x}\left[n\right]$ is the Kalman gain, given by:

(18) $$M_{x}\left[n\right]=P\left[n|n-1\right]C^{T}{\left(CP\left[n|n-1\right]C^{T}+R\left[n\right]\right)}^{-1}$$

where R[n] is the same as R mentioned earlier. Then, the error covariance is computed as:

(19) $$P\left[n|n\right]=\left(I-M_{x}\left[n\right]C\right)P\left[n|n-1\right]$$

Finally, the displacement $\hat{u}$ displacement estimated from the Kalman filter us given by:

(20) $$\hat{u}\left[n|n\right]=C\hat{x}\left[n|n\right]$$

An error correction factor α for the strain-based displacement is also applied to compensate for errors in the determination of the neutral axis location in the physical structure. The correction factor is described by Park et al. [39]. The relationship used to obtain displacement from strain is explained next.

#### 5.1.1. Strain-Based Displacement Estimation

A summary of the strain–displacement relationship required for the Kalman filter estimation is explained below [39]. Both quantities can be expressed as a linear combination of modes as given by:

(21) u=Φq

(22) ε=Ψq

where u and ε represent displacement and strain, Φ and Ψ are the displacement and strain mode shapes respectively, and q is the modal coordinates. The modal coordinates can be obtained using the relation:

(23) $$q=\Psi^{+}\varepsilon$$

where + denotes the Moore–Penrose pseudoinverse. By substituting Equation (22) into Equation (20), the strain-displacement relationship is obtained as:

(24) $$u={\Phi \Psi }^{+}\varepsilon$$

For the displacement mode shapes, the assumed modes method is used to obtain a reasonable approximation. However, for the strain mode shapes, the relationship between strain and curvature is used, knowing that curvature is the second derivative of displacement:

(25) $$\varepsilon =-u^{\prime \prime }y=-y\Phi^{\prime \prime }q$$

where $-u^{\prime \prime }$ and $\Phi^{\prime \prime }$ are the second spatial derivatives of displacements and displacement mode shapes while y is the neutral axis. Thus, the second derivative of the displacement mode shapes are used as the strain mode shapes. Essentially, the relationship between strain and displacement can be expressed as:

(26) u=De

where $D=\frac{1}{y}{\Phi \Phi }^{\prime \prime +}.$ In the next subsection, the performance of the earlier explained multimetric approach for displacement estimation is validated for its accuracy in both time and frequency domains. Note that for the displacement estimation approach employed, the accelerometer should be placed at the location where the displacement is being estimated, and the number of strain gages used should be at least the number of assumed modes considered. Care should also be taken not to place the strain gages at positions of anti-nodes in the modes of interest.

#### 5.1.2. Laboratory Validation of Multimetric Displacement Estimation

**Figure 9:**
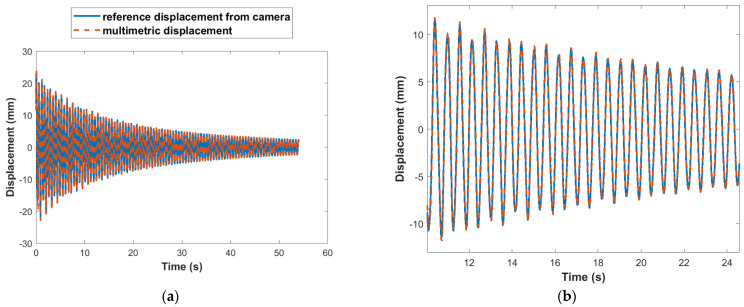
Comparison of estimated and reference first-story displacement. (**a**) Time domain comparison. (**b**) Time domain close-up view.

**Figure 10:**
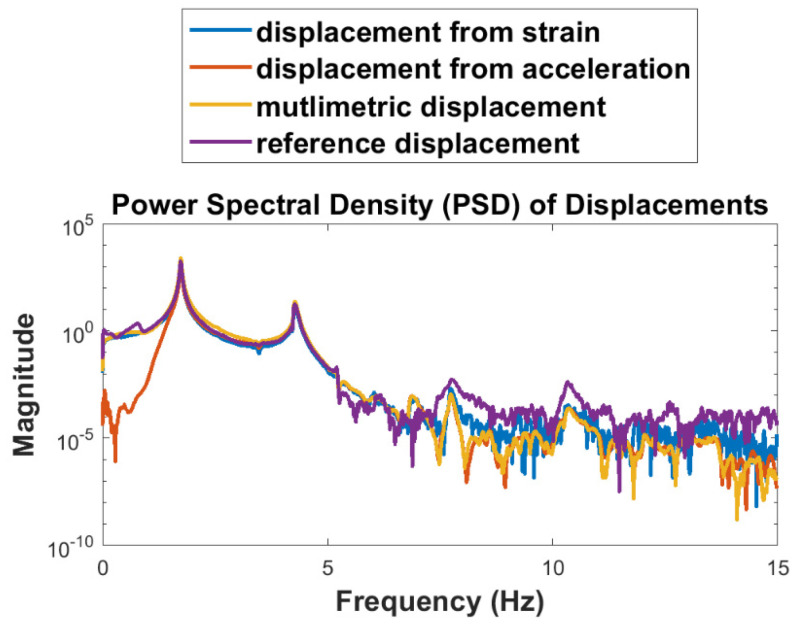
Comparison of estimated and reference first-story displacement in frequency domain.

The wireless strain sensor is employed to validate the multimetric displacement estimation of the test structure shown in Figure 7. For this study, two strain gages are attached to the structure, near the base and the first story, respectively. A stationary smartphone’s camera is used for the reference displacement measurement, which tracks the marker attached to the frame shown in Figure 7. The structure is again subjected to an impulsive load. The multimetric displacement estimation, as detailed in Section 5.1 is obtained using the synchronized free vibration response of strain and acceleration records from the Xnode attached to the test structure. A sampling rate of 100 Hz is used for the multimetric sensing. Figure 9a shows the results of the first-story displacement estimated using the multimetric approach with respect to the reference displacement in the time domain. The result shows good correlation between both estimates. A close-up view is shown in Figure 9b. Furthermore, Figure 10 compares the results in the frequency domain. Note that the acceleration-based displacement is unable to capture the low-frequency, pseudo-static displacement component. However, the strain-based and multimetric-based approaches are closer to the reference in that region, whereas in the higher frequency region, the displacement is well captured by the multimetric approach because of the use of acceleration data. In addition, note that the reference measurement using the camera was less accurate above 7.5 Hz due to pixilation. Therefore, while the acceleration-based displacement exhibits errors in the low frequency and the strain-based displacement is noisy in the higher frequency regions, the multimetric-based results perform well across the entire spectrum.

**Figure 11:**
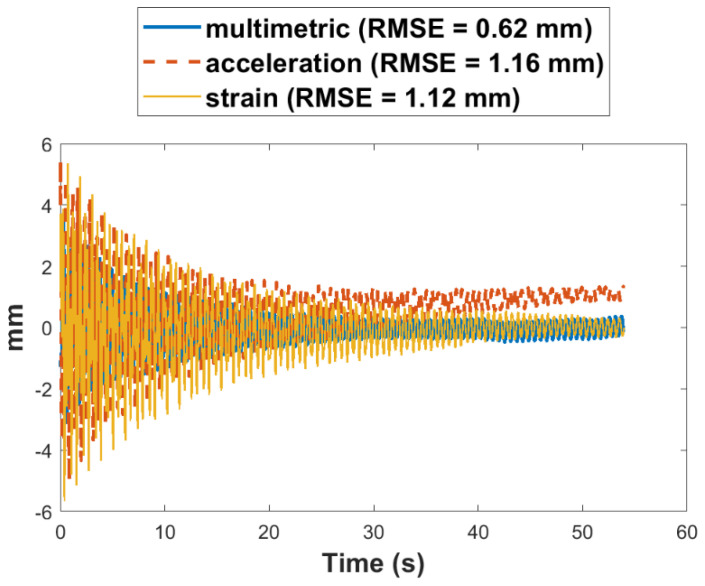
First-story displacement errors from different methods.

To further illustrate the advantages of the multimetric approach, Figure 11 and Table 3 compare the error metrics for different approaches with respect to the reference. First, a low pass filter with a cut off at 7.5 Hz is applied to all signals to remove the root–mean–square error (RMSE), together with the maximum absolute error (MAE) metric, were used for the comparison. The metrics are computed by:

(27) $$\mathrm{RMSE}=\sqrt{\frac{\sum_{i=1}^{N}{\left(y_{i}-{\hat{y}}_{i}\right)}^{2}}{N}}$$

(28) $$\mathrm{Maximum}\;\mathrm{Absolute}\;\mathrm{Error}=\max\left|y_{i}-{\hat{y}}_{i}\right|$$

Table 2 also shows the percentage improvement in RMSE of the multimetric method over the displacement based on only strain or acceleration for each test. An average reduction of 50% in RMSE and 45% in MAE were obtained using the multimetric approach. Next, an application of multimetric displacement estimation is illustrated using the Xnode for camera motion compensation.

## 6. Camera Motion Compensation

One of the major challenges in using computer vision (CV) for reliable displacement estimation is camera motion. CV based systems have been recently used to measure displacements in bridges, buildings, and lock gates [51,52,53,54,55]. Vision-based techniques can provide displacement measurements by tracking a region or point of interest on a structure. Generally, feature-based tracking methods are used for displacement estimation [56]. Therefore, this study uses the Kanade–Lucas–Tomasi (KLT) based feature tracking algorithm for displacement estimation [57]. Although, the accuracy of multi-metric displacement estimation has been verified in the previous section, vision-based displacement methods using the KLT algorithm have the advantage of estimating dense displacement fields of multiple points in the field of view (FOV). However, camera vibrations observed during field deployments due to external environmental conditions such as traffic, wind, and ambient noise can degrade intended measurement quality. Therefore, to obtain better measurement accuracy, methods need to be developed to minimize the errors induced by camera motion.

Several researchers have applied different motion compensation techniques [58,59,60,61]. For example, for vertical displacement measurement of a bridge, Chen et al. [60] compensated for only the vertical vibrational motion of a camera using a stationary reference in the background. The compensation was done with the assumption that the rotational motion was small. In such instances, when measurement only in one degree of freedom (DOF) is of interest, then compensating for only the corresponding DOF in the camera motion is sufficient. Essentially, for measuring the vertical deflection at a region of interest, compensating only for the vertical translation motion could be sufficient, provided the rotational motion is small and negligible. Indeed, nearly all of the previously developed motion compensation approaches rely on the presence of a stationary background feature being present in the scene. However, in practice, finding a stationary reference in the vicinity of the region of interest being tracked on a structure can be quite challenging.

This section proposes an approach for camera motion compensation using the multi-metric displacements. The developed multimetric sensing framework comprises of sensors with high sensitivity and good resolution, which enables the capability to be used to correct errors due to camera motion that may occur during vision-based measurements. The performance of the proposed method is then validated experimentally.

### 6.1. Multimetric-Based Camera Motion Compensation

A camera motion compensation approach without a stationary reference is proposed, leveraging the multimetric displacement data obtained using Xnode at a location. Herein, a pin-hole camera model is considered with a focal length f. Displacement measured at a distance Z from the camera can be obtained as:

(29) $${\hat{y}}_{\mathrm{cam}}=\frac{Zu_{\mathrm{cam}}}{f}$$

where ${\hat{y}}_{\mathrm{cam}}$ is the measured displacement in world-coordinates, and $u_{\mathrm{cam}}$ is the displacement in image coordinates. It is assumed that the camera vibration is primarily translational, and the rotational motion is negligible. The displacement in world-coordinates for a point in the FOV estimated using the multi-metric data is denoted by ${\hat{y}}_{\mathrm{multimetric}}$. Under the influence of camera vibration, the raw displacement of the point estimated using the camera is represented as ${\hat{y}}_{\mathrm{cam}\_\mathrm{vib}}$. Thus, the error due to camera motion in world-coordinates is obtained as:

(30) $${\hat{y}}_{\mathrm{cam}\_\mathrm{motion}}={\hat{y}}_{\mathrm{cam}\_\mathrm{vib}}-{\hat{y}}_{\mathrm{multi}-\mathrm{metric}}$$

Subsequently, the obtained camera motion error can be compensated to estimate the displacement of any other point (${\hat{y}}_{\mathrm{FOV}}$) in the FOV at a known distance from the camera as:

(31) $${\hat{y}}_{\mathrm{compensated}\_\mathrm{FOV}}={\hat{y}}_{\mathrm{FOV}}-{\hat{y}}_{\mathrm{cam}\_\mathrm{motion}}$$

thereby allowing the vision-based methods to estimate displacement elsewhere in the FOV without any stationary background features. This approach can prove to be useful when dense-field displacement measurements are necessary with only a limited number of sensors on a structure.

### 6.2. Laboratory Experiment for Camera Motion Compensation

**Figure 12:**
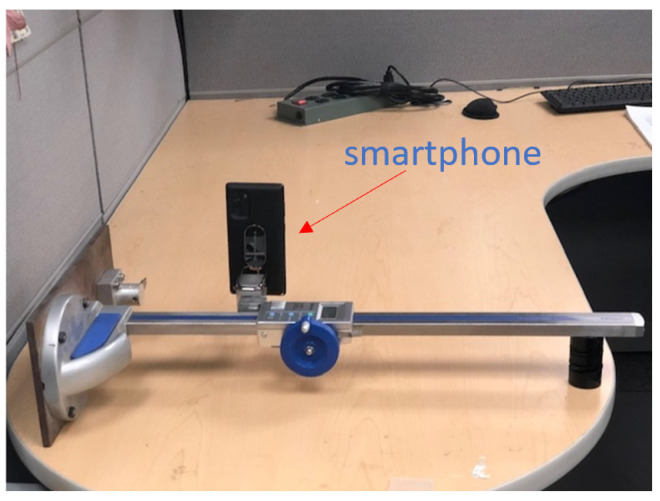
Linear rail set up for inducing translational camera motion.

For validation, a laboratory setup to use a camera to measure the free-vibration response of the two-story building frame model, shown in Figure 7. The displacement of first story is tracked by selecting salient features on the Xnode. A smartphone camera attached to a linear rail is subject to a random translational vibration motion, as shown in Figure 12. For reference, a stationary camera is used with the entire building model in the FOV. The first-story displacement is estimated using the multimetric approach with the Xnode.

**Figure 13:**
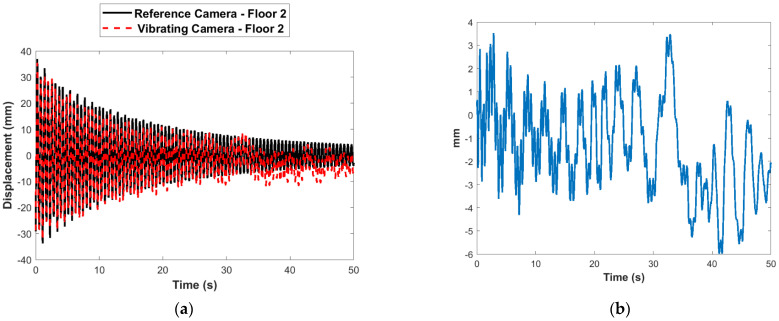
Comparison of estimated and reference second-story displacement: (**a**) in time domain before compensation of vibrating camera; (**b**) errors before compensation of vibrating camera.

**Figure 14:**
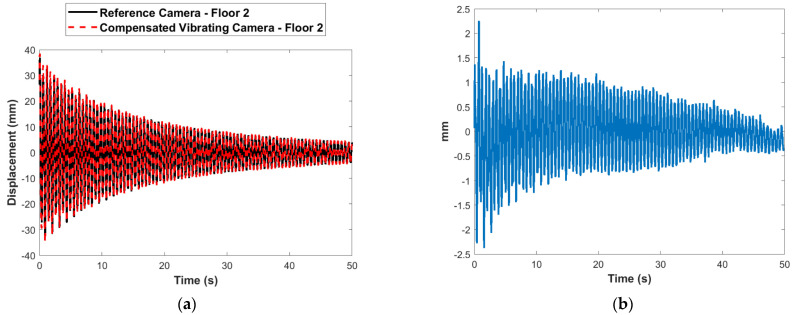
Comparison of estimated and reference second-story displacement: (**a**) in time domain after compensation of vibrating camera; (**b**) errors after compensation of vibrating camera.

The camera vibration is obtained using the multimetric first-story displacement measurement obtained from the Xnode as shown in Equation (30). The estimation of displacement at a point elsewhere in the FOV (the second story) is evaluated by compensating for the estimated translational camera motion as in Equation (31). Figure 13a compares the raw displacement estimated at the first floor using the vibrating camera with the reference measurement. The error before compensation with respect to the reference is shown in Figure 13b. Figure 14a shows the comparison of the estimated second-story displacement from the vibrating camera after applying the correction to the reference measurement. The error after compensation is shown in Figure 14b. The RMSE obtained, in this case, is 0.67 mm. Again, the results match very well with the stationary reference camera.

**Figure 15:**
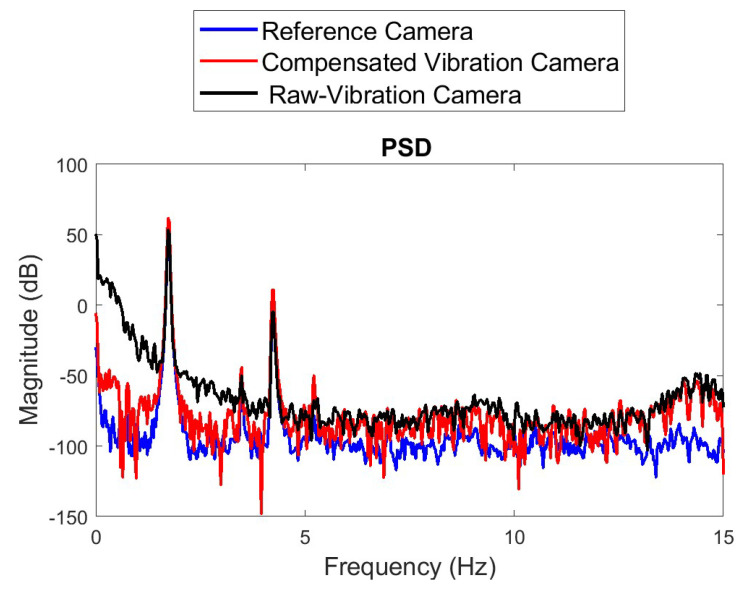
Comparison of estimated and reference second-story displacement in frequency domain.

Figure 15 shows the frequency domain comparison of the estimated second-story displacements from the reference camera with those from the vibrating camera before and after motion compensation. The results show much better agreement with the reference measurements in the frequency domain after the camera motion was applied. The performance of the compensation under high level of noise was also investigated. By introducing white noise consisting of 10% signal to noise ratio into the original signal, the RMSE was found to be 1.83 mm. However, due to the high-quality of the hardware and the precision of the 24-bit ADC, the experimental noise levels found in the sensors considered in this paper were in the range of 0.1% to 1%.

Overall, the proposed setup allows for camera-motion compensation to obtain the corrected displacement estimate at any other point in the FOV, as was demonstrated for the second-story displacement estimate. Thus, the setup enables reliable multi-point displacement estimation using vision-based measurement systems.

## 7. Conclusions

In this paper, a framework for multimetric sensing of strain and acceleration was developed using the Xnode wireless sensing platform. First, to enable multimetric sensing, a new strain sensor board was designed and integrated with the existing accelerometer sensor hardware on the Xnode platform. Subsequently, a software algorithm was developed to acquire strain signals with acceleration on the Xnode. To validate the efficacy of the proposed framework, the accuracy of the strain circuit was first validated experimentally for both static and dynamic strain measurements using a laboratory setup consisting of a simply supported cantilever beam and a two-story building model, respectively. Furthermore, the application of the proposed multimetric sensing framework was demonstrated for total displacement estimation on the two-story model in the laboratory. A Kalman filter was used to fuse strain and acceleration measurements from the Xnode, and estimate accurate displacement. The obtained results were compared with reference obtained from a stationary camera and, the RMSE was found to be 0.57 mm. Additionally, the framework is integrated with a camera measurement system by leveraging the multimetric displacements to compensate for camera motion errors that may occur when carrying out vision-based measurements, thus obtaining reliable multi-point displacement estimates without a stationary background reference. The proposed approach was validated in the laboratory by compensating the errors from camera vibration induced on a smartphone camera, obtaining an RMSE of 0.66 mm with respect to the reference. Overall, the results demonstrate the significant potential of the developed framework for high fidelity multimetric sensing.

## Figures and Tables

**Table 1:** Properties for static strain verification.

Property	Value
Length of steel beam	11 in
Thickness of steel beam	0.048 in
Strain gage location	3 in from fixed end
Weights used	27.4 g, 47.1 g and 97.3 g
Weight location	1 in from free end

**Table 2:** Experimental results for static strain verification.

Weight (g)	Theoretical (με)	Xnode (με)
27.4	35.92	36.51
47.1	62.85	63.21
97.3	134.7	136

**Table 3:** Comparison of estimated first-story displacement.

Test	Root–Mean–Square Error (mm)				Maximum Absolute Error (mm)			
	Strain	Acceleration	Multimetric	% Improvement	Strain	Acceleration	Multimetric	% Improvement
1	1.37	2.25	0.71	48	6.40	7.47	3.46	46
2	1.12	1.16	0.62	45	5.42	5.37	3.70	31
3	0.90	2.00	0.38	58	4.86	6.82	1.99	59
Average	1.13	1.80	0.57	50	5.56	6.55	3.05	45
